# A Qualitative Study on the Benefits and Costs to Creators and Producers of Medical Open Educational Resources: Freely Given, but at What Cost?

**DOI:** 10.7759/cureus.111307

**Published:** 2026-06-22

**Authors:** Parnian Pardis, Avital Pitkis, Tanya Horsley, Bhargavi Dhanireddy, Michael Gottlieb, Brent Thoma, Teresa M Chan, Matthew D Zuckerman

**Affiliations:** 1 Michael G. DeGroote School of Medicine, McMaster University, Hamilton, CAN; 2 Family Medicine, McMaster University, Hamilton, CAN; 3 Research Unit, Royal College of Physicians and Surgeons, Ottawa, CAN; 4 Obstetrics and Gynecology, Carle Health, Urbana, USA; 5 Emergency Medicine, Rush University Medical Center, Chicago, USA; 6 Emergency Medicine, University of Saskatchewan, Saskatoon, CAN; 7 Emergency Medicine, Toronto Metropolitan University, William Osler Health Systems, Brampton, CAN; 8 Emergency Medicine, University of Colorado School of Medicine, Denver, USA

**Keywords:** cost, educational sustainability, faculty advancement, open-access medical education, open educational resources, teaching in emergency medicine

## Abstract

Background

The Free Open-Access Medical Education (FOAM) movement has produced a wealth of open educational resources (OERs) for asynchronous learning, bedside teaching, and journal clubs. Although these resources remain free to end-users, the burden of production and cost of maintenance has largely fallen on their producers. Much of this work is sustained through the unpaid and unfunded efforts of creators, contributors, and producers.

Methodology

This qualitative study was conducted within a constructivist and interpretivist paradigm, using semi-structured interviews. Website administrators from the top 25 Emergency Medicine FOAM platforms, identified via the Social Media Index, were invited to participate. One-on-one semi-structured interviews focused on motivations, challenges, and sustainability were recorded and transcribed verbatim. A diverse research team collaboratively coded transcripts through thematic analysis, refining a framework of themes and subthemes. Data collection concluded upon reaching thematic saturation.

Results

A total of 11 website administrators were interviewed, with interviews lasting between 14 and 48 minutes and generating 125 pages of transcripts. Thematic saturation was achieved with these participants. Participants (aged 35 to 63 years) described many benefits of their OER involvement, including enhancing their own learning, personal fulfillment, and career advancement. However, they also reported significant barriers to sustainability, such as financial costs, opportunity costs (e.g., reduced time for personal activities), and mounting pressure to consistently produce content.

Conclusions

The nomenclature FOAM does not equate to entirely free. The costs are shifted away from end-users, and while there are clear benefits to the model, the long-term sustainability of the movement is uncertain, particularly in the absence of sophisticated funding models. It is crucial to examine how we support this movement as a broader medical education community to determine how we might continue to foster a widely useful and democratizing approach to learning.

## Introduction

Open educational resources (OERs) are increasingly valued in academic circles [1]. In medical education, OERs are most commonly realized through the Free Open-Access Medical Education (FOAM) movement, which disseminates openly accessible educational content used for asynchronous learning [2-4], on-shift learning [5], and journal clubs [6,7]. Across several specialties, FOAM-based resources are being incorporated into formal curricula [8] and proponents are advocating for it to be used in academic promotion [9-11]. However, the literature presents conflicting trends, with evidence of both a substantial increase in medical OER creation [12,13], as well as a decline in the number of active medical OER resources over time [14]. This apparent contradiction raises important questions about the lifecycle of FOAM resources and the long-term sustainability of medical OER initiatives.

By its nature, OERs are accessible to end-users without the use of any paywalls. While free to consumers, the costs of development, production, and maintenance now fall on the entrepreneurs who create and distribute these educational resources. This work has historically relied upon the unpaid and unfunded time of contributors [13]. Despite this perceived burden, what motivates creators to enter this enterprise, along with their perceived costs and returns, has not been explored deeply.

We conducted a qualitative study to examine why FOAM creators initiate resource development; what personal, professional, and economic costs they incur; and what forms of “return on investment” (ROI), including scholarly recognition, career advancement, personal fulfillment, and community impact, they perceive from their involvement. We examined these benefits and costs both as distinct considerations and as interrelated experiences that shape creators’ engagement over time. In addition, we sought to identify factors that influence creators’ decisions to continue or discontinue production over time. Through this analysis, we aimed to generate a conceptual framework describing the motivational arc of FOAM production; this framework emerged from the data and is presented as an explanatory model rather than an a priori hypothesis. We anticipate that these findings will inform strategies to improve the long-term sustainability of FOAM and other medical OER initiatives.

## Materials and methods

Our research was situated within a constructivist and interpretivist paradigm, recognizing that individuals construct their understanding of reality through self-experience and reflection. Within our interview-based study, we analyzed transcripts using interpretive description methodology to extract elements that became the themes for our paper [15]. The Standards for Reporting Qualitative Research informed the reporting of our research [16].

Researcher characteristics

Our research team was diverse and consisted of four experienced academic physicians (MZ, MG, BT, TC), one business-trained academic administrator (TH), one non-clinical medical faculty member (YY), and three medical trainees (PP, BD, AP). Two members of the research team (YY, TC) have received formal training in qualitative research methods, and three had previous relationships with several participants (MZ, TC, BT). Four of the researchers identify as males, and five identify as females. During analysis, team members’ differing professional roles and career stages informed interpretive discussions, with clinicians contributing contextual insight into academic and FOAM ecosystems and non‑clinical members and trainees highlighting workload and opportunity cost.

Participants

Based on the Social Media Index [17,18], we conducted intentional sampling from a list of the top 25 FOAM websites for emergency medicine and critical care to include those individuals most active in this space. Site administrators were chosen as potential participants, assuming their knowledge of administrative and operational aspects, as well as their contributions to the educational content. Site administrators were identified clearly on the site pages and invited electronically via email or their website to participate in semi-structured interviews.

Ethics

The Hamilton Integrated Research Ethics Board granted ethics approval for our research study (approval number: HIREB-11529). All participants consented to a recording of their interviews. No confidentiality or security issues were identified during participant interviews.

Data collection

Each participant was involved in a semi-structured interview using prompts from the interview guide with one of three members of the research team (PP, MZ, BD) (Appendices). All interviewers were trained by the principal investigator. Pilot interviews were conducted with three participants to ensure adequate interview flow. All interviews were conducted and recorded using Zoom (Zoom Video Communications, Inc). Audio files were transcribed by a trained medical transcriptionist (EC) who anonymized interviews and removed any identifying information. We did not solicit participant review of transcripts, but all participants affirmatively responded to email communication if verification was required. We did not record any field notes during the interview.

Data analysis

Transcripts were analyzed by five authors (PP, TH, MZ, BD, YY) using thematic analysis [15,19]. Line-by-line, two transcripts were coded together by the authors to inductively develop a framework of themes and subthemes intended to guide the analysis of subsequent transcripts. Researchers subsequently coded transcripts, adding codes to the existing framework when applicable. Codes were developed inductively and iteratively refined through collaborative team discussions, with attention to patterned meanings across participants rather than frequency counts. Analytic decisions, including code refinement, theme boundaries, and conceptual relationships, were reached through consensus and documented in the audit trail, allowing themes to evolve as interpretations deepened over time. Coding was discussed deeply among the team during multiple meetings over six months to explore alignment, resolve any discrepancies, and achieve consensus. This systematic approach was supported by an audit trail generated on Google Docs (Alphabet Incorporated, Mountain View, CA, USA) during transcript analysis. Thematic saturation was determined through iterative team discussions when no new themes or conceptual insights were identified across successive interviews. Once we achieved thematic saturation, we concluded data collection and analysis. Pilot interviews were not included in the data analysis. All codes were attached to specific quotations by interview participants. Once all transcripts were analyzed, codes were reorganized where appropriate to illustrate both minor and major themes and inform our final interpretation.

## Results

A total of 11 website administrators were interviewed between March and December, 2021. The sample included two individuals who self-identified as female and nine as male, and ranged in age from 35 to 63 years. The duration of the interview ranged between 14 and 48 minutes. Verbatim transcription yielded 55,047 words. The overall motivational arc of OER producers, defined as a path of entry, maturation, and withdrawal, is summarized in Table 1.

**Table 1:** Summary of the motivational arc of OER content producers.

Stage	Theme
Entry	Contributing to innovation in medical education, social media, and patient care
	Networking
	New opportunities for scholarship, employment, and personal learning
	Hobby
Maturation	Opportunities for scholarship, employment, and personal learning
	Impact on patient care
	Sense of community
	Learning knowledge and skills from others in the community
	Mental health benefits
	External recognition and user engagement
	Leaving a legacy and impact on the specialty
	Being able to reinvest income into the platform and maintain sustainability
Withdrawal	Less time for other academic and professional pursuits
	Impacts on family and relationships
	Difficulty maintaining work-life balance
	Lack of feedback regarding the impact of content on clinical practice
	User criticisms
	Pressure to ensure content is always up to date and error-free
	Conflicts with affiliated institutions
	Financial difficulties and concerns with sustainability
	Increasing costs

Hobby time

A hobby is defined as “a pursuit outside of one’s regular occupation engaged in especially for relaxation” [20]. It can be associated with enjoyment, desire for mastery, and alignment with identity. Many FOAM creators similarly view content creation as a hobby. One person commented:

“…I think … for the amount of time that I spent with it and how much pleasure I get out if it I would probably best describe it as a hobby … I don’t mean this as a casual hobby I mean like as something that I find pleasure in, and I enjoy improving at. Yeah, I find this really weird internal motivation with it … I wonder what like a regular person does, because whenever I have free time I am doing [this] stuff because I actually enjoy it” (Participant 10).

While intrinsic rewards may result from engaging in hobbies (e.g., the sale of excess pottery made in a home kiln), they are not generally the primary aim. Creators, often situated in traditional academic settings, also note associated opportunities for networking, scholarship, employment, and personal learning that come with this hobby. One creator referred to it as: “…a hobby with benefits” (Participant 3).

As creators transition from an “enthusiastic hobby” to a more professionalized enterprise, parallel concerns and dissatisfaction often emerge. When the need for continued growth or long-term sustainability becomes apparent, particularly in relation to financial, opportunity, or human resource costs, creators may be at increased risk of disengagement. In response, some reduce their level of involvement, while others exit the space entirely.

Money matters: monetary costs and benefits

Participants shared perspectives that while “free medical education” is being provided on FOAM platforms, it comes with a price tag for its producers. Of those interviewed, few drew on external sources of funding; one mentioned having conference funding, and another had funding from an education and research institute. In the absence of formal funding, the most common methods of generating a source of revenue were commonly advertisements, paid content (such as paper summaries and additional courses), merchandise, sponsorships, and/or donations. While perceived as necessary for sustainability, the majority of participants were not keen on introducing these practices to their platforms, instead wanting to emphasize the free and openly accessible nature of their content.

Most creators reported being able to generate an income; however, nearly half of the participants recounted struggling to generate enough for a cost-recovery model. In fact, yearly costs to require recovery ranged between $5,000 and $62,000. Furthermore, nearly half of all participants self-funded their platforms for various periods of time, resulting in a financial loss. For instance, one person commented: “...I am probably [$5000] to $8000 in the hole, net negative that I am still paying. But it is not [$]20,000 anymore. …I am [still] paying quite a bit of money out of my own pocket…” (Participant 2).

While a few individuals were able to make a net profit using FOAM, for instance, by creating an online course and a question bank, most expressed not being able to generate a net profit. Most participants stated that they reinvest any financial gains back into their websites to maintain and grow operations. For instance, one participant remarked: “…it doesn’t make a [lot] of money, but it makes enough to at least sustain the site. And then any positive stuff it allows me to build like an app as well to put money into that” (Participant 8).

The sustainability of FOAM efforts and website maintenance was a frequently cited concern in light of the monetary aspect, as one participant commented: “...I have kind of beat the door so hard that I can’t seem to break a certain threshold of making significant profit…why am I wasting so much time trying to hustle for money when I could just maybe step it back to a level that I feel comfortable with” (Participant 1).

Additionally, with pressures to maintain a constant stream of content and continue to grow their platforms, many reported debt, financial difficulties, and struggles to recover costs as expressed by: “But now it is a debt eschewing hole of blackness on most occasions which I am scrambling to stop hitting the bottom on. And just trying to work my way out” (Participant 6).

Another participant emphasized the unexpected nature of these financial challenges: “…the only negative is just … the salary cut that I had not anticipated during my career. I always thought it was going to … grow slowly over time as you promote it. And it’s like half … And then I spent so much time trying to get up to that differential that I lost even more time to work on [Platform] itself” (Participant 1).

To avoid a negative balance, the amount of work required can often be unsustainable: “I think anybody doing FOAM activities will 100% realize that it is a horrible cost-benefit analysis. We are all losing a … ton of money…. There may be some people who actually if you look at the spreadsheet are a little bit positive but that doesn’t account for the hours. And our hourly rate…” (Participant 4).

Moreover, many voiced their worries about increasing debt load with rising costs of website maintenance and content production, especially as they expand their sites, hire more content/operational contributors, and increase server space. One participant voiced: “What I have realized is as the site has gotten bigger, as we have more contributors, as I required more or server space and other types of things the amount that I pay per year has gone up extraordinarily” (Participant 2).

However, the participants who were able to generate profit reported that this was motivating and energized them to continue in the FOAM space: One participant remarked: “...from the business side of things, it is becoming more sustainable, and we are actually starting to make some income off of it. But it made me … more motivated to create more content” (Participant 8). Another highlighted the following: “...I really hope it…keeps going because that will help motivate us to create more free content because we assume that the free content drive is to the paid content” (Participant 10).

Financial debt, increasing costs, financial uncertainty, and opportunity costs appear to be stressors, and concerns regarding sustainability were frequently mentioned and cited as a barrier to increasing involvement in the OER space. While financial gain was mentioned only once as a motivating factor for involvement in the OER space, several individuals reported that it helped content producers continue their work in this area.

Beyond the blog: opportunity costs and benefits

Despite the financial burdens and stressors, a common viewpoint held by all those interviewed is that engaging in OER afforded countless advantageous opportunities. The majority of participants affirmed that being involved in OER has given them the opportunity to function as innovators in the fields of teaching, social media, and medical education by filling perceived, unmet needs of the medical community.

For instance, two participants explained that during a time of skepticism about the utility of social media, OER was an opportunity for them to improve social media platforms and surpass traditional academics by creating educational resources that have unprecedented accessibility to audiences.

Many found that traditional whiteboard and PowerPoint presentation-style medical education may not meet the needs of all students, and, therefore, OER allowed them to make an impact on medical education by implementing novel strategies, such as an online case library. Some additionally expressed having a main goal of influencing patient care and changing clinical practice, which was facilitated by OER, such as this participant: “…you need to be able to recognize the needle in the haystack when you see those EKGs that show occlusion. And so, to me it is by far the most important problem in emergency cardiology. And that is why I have been working on it for my whole career” (Participant 7).

Additionally, several participants expressed that being in the OER community afforded them remarkable networking opportunities, making global friends and connections with common interests. These new connections opened the door to new collaborations, leading to further opportunities in scholarship, employment, and individual learning. These new collaborations on research projects and other academic activities that emerged were attributed specifically to engaging in OER communities, therefore, leading to a substantial increase in academic outputs such as publications, abstracts, and speaking opportunities. Participants discussed having numerous conference presentations and lecturing opportunities directly in response to their involvement in OER and connections to other scholars. To several of our participants, OER became a dynamic alternative to traditional academia. It became an avenue for them to fulfill their desire to teach without the difficulties associated with traditional forms of academia: “And I think the reason that I went into academics was because of teaching and education and working and mentoring people. And what I found is that the further along I matriculated into the ladder of promotion and tenure and taking on different positions, the less I could teach because I was in more meetings. I was working less clinically. I had more forms and checkboxes that I had to fill. And I feel like [Platform] has been such a nice way to continue teaching, continue mentoring and doing all of the things that I am passionate about without having all of the academic checkboxes that are required when you are in academics” (Participant 2).

Several creators described feeling motivated by positive responses to the work they were engaging in, including feedback, external recognition, and user engagement. Many expressed high levels of satisfaction seeing their “impact” through analytics, especially as they may not have been able to reach that level of exposure with more traditional teaching and scholarship methods. As one participant stated: “And I wrote a post which got a 140,000 views in the first hour. It went on the number one post for Google, and I was so excited. I was just thrilled …. There are people out there that want to read about things. And that really stimulated me to write posts” (Participant 6).

Meanwhile, another participant shared similar impressions: “...we are getting almost like 50,000 page views a month. And it is like wow I can reach a lot more people … Versus I think that our research I think I thought maybe one you know every few months. Like I published a bunch of papers on ultrasound, but I didn't get nearly as much engagement as I do now with actual people using it” (Participant 8).

One participant added that continuing their legacy and feeling that they have left an impact on their specialty held a lot of meaning for them as well.

Furthermore, we noted that participants expressed valuable academic opportunities; several participants discussed that OER brought them new professional opportunities as well. Examples include working less in the ED and focusing on OER, traveling while creating content, and involvement in new professional committees. OER also helped many individuals progress their careers to become professors and obtain other competitive positions. One participant recounted: “I am a professor and the only reason … is because of [Platform] … it is the pretty much sole thing that has accelerated my academic career” (Participant 9).

The participant additionally remarked upon how the appeal of FOAM was the commitment required to stay up-to-date and the ability to learn by teaching. It led them to read more papers, stay up to date, and learn independently to create their educational materials: “I just wanted to be the best as I could as an emergency doctor so I wanted to learn as much as I could. And there was no better way to learn yourself than to teach” (Participant 9).

Others shared that they were able to learn a variety of knowledge and skills from others in the FOAM community, including website management skills, leadership skills, and new medical information. The following are two typical quotations that describe how their journey through the world of FOAM led them to develop their skills: “...it was clear that there was a community, and not just a community but a community with shared values, but it was also clearly changing people’s practice. I was learning from them, and they were learning from me … It was more about actively participating in that shared community, so that we all continuously make each other better...” (Participant 4). “I have learned so much from other people, in terms of how to be a leader, how to be a team player. Learning from other mistakes. And being a better doctor because of it. And through the models of other people from other places and different healthcare systems it has really pushed me to be better version of myself. Not only as a physician but also as a person” (Participant 2).

Overall, being in this dedicated community and collaborating with like-minded individuals increased educational, scholarship, employment, and learning opportunities, which participants expressed as a significant motivator to continue producing more FOAM content.

However, the majority of interviewees also commented that involvement in FOAM comes with important opportunity costs, i.e., time. Given the amount of investment of time required to create and maintain content and upkeep platforms, participants reported needing to cut down on clinical time and decreasing involvement in other academic activities. Creators noted having less time to spend on leisure activities, as well as strong (sometimes negative) impacts on family and relationships, with one person disclosing that FOAM involvement was a large contributor to the breakdown of an important relationship. As one participant explained succinctly: “The cost is time … cost is purely time” (Participant 11).

The #FOAM life: personal costs and benefits

Participants found benefits to their personal lives and mental health as part of being involved in a dedicated community and learning from one another, as was described by Participant 4 in the previous section.

However, involvement in FOAM was not universally seen as positive. Many participants noted the dark side of being engaged in this activity. Most notably, many highlighted how there is a perceived pressure to produce, maintain, and create, which was seen as a source of stress for its creators. One commonly reported issue was difficulty maintaining work-life balance; participants explained that their work in FOAM created obstacles in relationships and difficulties maintaining a personal life: “...the biggest things for me were finding a balance between doing the stuff online and not losing a sense of like your own personal life. Like when you are on website, or you are on social media it is like you are on your phone or on your tablet it is on your desktop, and you are just always getting pinged about things” (Participant 2).

One participant added that with the significant amount of work needed to create content, it becomes difficult to stay engaged if there is a lack of user feedback regarding the impact of the materials on clinical care and practice.

On the other hand, many participants stated that user feedback and criticism can be their own additional stressor. For instance, having a global audience means that there is a risk of offending users if certain cultural intricacies or details are not known. Some even reported having users write personal attacks toward them, disparage their credentials, and even troll.

Moreover, there was a lot of pressure voiced by several participants to have new content regularly available to prevent audience disappointment. Several individuals also felt pressure to ensure that their information is free of errors and is continuously up to date, due to the level of responsibility that their content carries: “...now I am … responsible for thousands of emergency providers around the world for … making sure that I am giving them … at least the closest thing to the truth that I can” (Participant 9).

Two participants described challenges related to maintaining professional independence. They reported conflicts with their host institutions over content ownership, intellectual property management, and the publication of controversial material, such as COVID-19 scientific data that contradicted institutional guidelines. In these situations, the perceived risk of career repercussions was high, requiring creators to exercise additional caution to continue their work in OER.

## Discussion

In our qualitative study, we describe a conceptual depiction of FOAM developers’ experiences, derived from participant narratives, that illustrates common patterns of entry, continued engagement, and disengagement for some producers. While not all producers have experienced the full arc, there are common experiences that allow important insight into Lin et al.’s findings that the number of active resources was decreasing [14]. Positive and negative factors described under the themes “monetary,” “opportunity,” and “human” suggest some aspects of OER production that could be leveraged to support producers.

While there are numerous studies exploring the experience of OER users [2-4], studies of producers are uncommon. This study is an important contribution to the literature as it helps provide insight into their experiences. While there are multiple factors that may influence initial entry into the OER realm, our results demonstrate the incredible challenge of working in this space over a prolonged period of time. Notably, the positive reasons for making OER contributions appear most valuable to junior faculty. In particular, the “opportunity” and “human” factors could be viewed as very appealing to those at the beginning of their career, hoping to “make their mark.” Several participants described perceiving that, as their careers progressed, the balance between perceived benefits and costs shifted in ways that made continued FOAM participation more challenging, though experiences varied by individual context. At the same time, their following is likely to be larger due to their early work, raising their visibility, with the resultant pushes for both more content and a higher risk of content inaccuracies or errors. This raises issues for the field because these senior members of the OER community often have years of know-how and experience that could be lost as they disengage [13].

Our findings offer a participant‑informed explanatory lens that may help contextualize previously observed declines in the number of active FOAM resources, without implying a causal or movement‑wide explanation [14]. The motivational arc presented here should therefore be understood as an interpretive framework reflecting shared patterns across participant narratives, rather than a definitive or predictive model of FOAM engagement. Similarly, if there is both a financial and human cost to engaging OER, this may explain why survival has led to groups amalgamating and joining forces (e.g., PulmCrit/EMCrit, CanadiEM) [21] to reduce the burden on individual producers. OER, like other types of academic work, would benefit from mentorship pathways and pipelines that support the development of new leaders. Strategies that have been effective in retaining and supporting academic clinicians in other contexts may have parallels in OER [22,23]. Other approaches to the development of these structures and systems to support the educational use of social media within institutions may also warrant further exploration [24]. In particular, acknowledgement of the contributions of OER creators in academic promotion [9-11,25] would better align OER production with other forms of scholarly productivity. Unfortunately, as these resources are often established outside of the academic ecosystems, these incentives will not work for all producers [26].

Limitations

Our study has several limitations. First, our enrollment strategy focused on top‑rated, currently active FOAM sites; therefore, the perspectives of less successful creators, individuals managing smaller platforms, or prior creators of defunct sites may not be fully represented. Second, several members of the research team had prior expertise in OER, which may have introduced pre‑existing interpretive frameworks. To mitigate this potential bias, interviews were conducted and transcribed by individuals separate from the analytic team and were anonymized before analysis. In addition, analytic decisions and reporting were undertaken collaboratively by team members with diverse professional roles, career stages, and levels of experience with OER, allowing multiple perspectives to be surfaced and debated during interpretation. As with most qualitative research, the findings are intended to provide conceptual insight rather than statistical generalizability, with transferability dependent on readers’ assessment of contextual alignment. Accordingly, these findings should be understood as informing discussion and hypothesis generation regarding FOAM sustainability, rather than as a validated or comprehensive account of the experiences of all OER producers.

## Conclusions

Our study explored the experiences of OER producers and identified various factors that impact their business lifecycle in terms of generating open educational resources. While their substantive enabling advantages to being a member of the OER movement, leadership in this movement has certainly exacted a toll on its contributors as well. We hope that this study provides some insight into the mindset of leaders within the OER movement. Participants commonly expressed uncertainty regarding the long‑term sustainability of their FOAM efforts, particularly in the absence of stable funding models and institutional support.

## Appendices

Interview guide

Why did you get into the FOAMed space originally?

What motivated you to become involved in this bigger FOAM movement as a major contributor? Or did you work pre-date this label?

Considering your original motivations and intentions, do you feel you’ve achieved or realized them?

What are the driving forces that continue to motivate you to maintain the website? Have these changed over time?

Talk to us about your organization - <INSERT NAME OF OUTLET>. How would you describe this entity (business, hobby, scholarship/research activity, etc)? Has this changed over time?

Now we will venture into speaking about the impact of FOAM on your life.

How has being involved with FOAM impacted your academic or professional life?

Pros: name recognition, promotion, speaking opportunities.

Cons: unrecognized contributions, decreased academic output, negative publicity, and negative feedback from peers.

How has being involved with FOAM impacted other aspects of life?

Pros: new relationships, financial.

Cons: time away from family/friends, financial.

Taking all of this into account, would you say that your investment has been worth it?

Do you have cost and revenue projections going forward?

Would you consider monetizing your content either through subscription or ad-based revenue if you do not currently?

If so, would your motivation for this monetization be cost recovery or income? What would you spend surplus income on (e.g., reinvestment into the site/people’s personal income to compensate for salary, to grow a company)?

In the era of the pandemic, how was your organization impacted?

Do you have any additional comments regarding the cost/benefit of your FOAM activities?
